# Impact of Cardiovascular Specialty Care Based on the Humanistic Concept on Medication Adherence, Glycolipid Metabolism Parameters, and Quality of Life in High‐Risk Cardiovascular Patients With Lipid Management

**DOI:** 10.1002/clc.70290

**Published:** 2026-06-15

**Authors:** Min Wang, Yonghui Xu, Huina Wang, Shurui Dou, Jingjing Xu, Fanfan Wang, Dan Hong

**Affiliations:** ^1^ Cardiology Department China‐Japan Friendship Hospital Beijing China; ^2^ Department of Cardiovascular Ⅱ, The First Aftiliated Hospital The First Clinical Medicine School of Guangdong Pharmaceutical University Guangzhou China

**Keywords:** cardiovascular specialty care, high‐risk cardiovascular disease, humanistic concept, lipid management, medication adherence, quality of life

## Abstract

**Objective:**

To investigate the effects of cardiovascular specialty nursing based on the humanistic concept on medication adherence, glycolipid metabolism parameters, psychological status, self‐management ability, and quality of life in high‐risk cardiovascular patients undergoing lipid management.

**Methods:**

In this retrospective study, 115 high‐risk cardiovascular patients admitted between December 2023 and November 2024 were selected from the medical record system and divided into two groups: a control group (*n* = 55, conventional care) and an observation group (*n* = 60, humanistic concept‐based specialty nursing). Outcomes compared included medication adherence assessed by the Morisky Medication Adherence Scale (MMAS‐8), glycolipid metabolism parameters including glycated hemoglobin (HbA1c), fasting plasma glucose (FPG), 2‐h postprandial glucose (2hPG), triglycerides (TG), total cholesterol (TC), and low‐density lipoprotein cholesterol (LDL‐C), psychological status assessed by the State Anxiety Inventory (S‐AI) and Trait Anxiety Inventory (T‐AI) of the State‐Trait Anxiety Inventory (STAI), self‐management ability assessed by the Exercise of Self‐Care Agency Scale (ESCA), and quality of life assessed by the Generic Quality of Life Inventory‐74 (GQOLI‐74).

**Results:**

The observation group demonstrated significantly higher total medication adherence (95.00% vs. 81.82%, χ² = 4.973, *p* < 0.05). Post‐intervention, all glycolipid metabolic indicators (HbA1c, FPG, 2hPG, TG, TC, LDL‐C), as well as S‐AI and T‐AI anxiety scores, were significantly lower in the observation group compared with the control group (*p* < 0.05). Additionally, the observation group showed significantly higher scores in all ESCA self‐management domains (self‐concept, self‐care responsibility, self‐care skills, and health knowledge) and across all GQOLI‐74 quality of life dimensions (material life, somatic function, social function, and psychological function) (*p* < 0.05).

**Conclusion:**

Cardiovascular specialty care based on the humanistic concept was associated with improvements in medication adherence, glycolipid metabolism parameters, psychological status, self‐management ability, and quality of life in high‐risk cardiovascular patients, suggesting high clinical applicability.

## Graphical abstract

This retrospective study of 115 high‐risk cardiovascular patients demonstrated that humanistic concept‐based cardiovascular specialty nursing significantly improved medication adherence (95.00% vs. 81.82%, *p* < 0.05), glycolipid metabolism (HbA1c, FPG, 2hPG, TG, TC, LDL‐C), anxiety levels (S‐AI and T‐AI), self‐management ability (ESCA), and quality of life (GQOLI‐74) compared with conventional care.

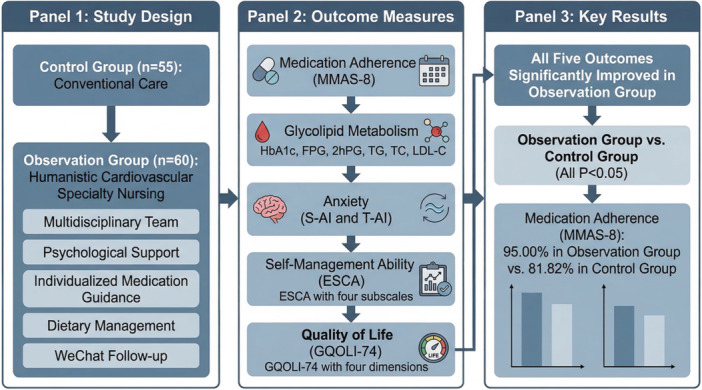

## 1 Introduction

Cardiovascular disease (CVD) encompasses a broad spectrum of circulatory system disorders, including ischemic heart disease, stroke, cardiomyopathy, hypertensive heart disease, and arrhythmias. Established risk factors such as smoking, dyslipidemia, diabetes mellitus, and obesity contribute substantially to CVD development, making it a leading cause of mortality worldwide [1, 2]. With population aging and lifestyle changes, CVD morbidity and mortality continue to rise, posing significant challenges to public health systems globally [3]. High‐risk cardiovascular patients—defined as individuals with three or more concurrent CVD risk factors (e.g., hypertension, diabetes, dyslipidemia, smoking, obesity, family history of premature CVD) or those with established atherosclerotic disease and a predicted 10‐year cardiovascular event risk exceeding 20% based on validated risk assessment tools—represent a particularly vulnerable population requiring intensive treatment and comprehensive management [4]. As Haidar A et al. [5] noted, these patients often require long‐term multi‐drug regimens, and concerns about medication side effects frequently compromise adherence, thereby affecting glycolipid metabolism control and diminishing both treatment outcomes and quality of life. Therefore, effective management and nursing care for this population is of paramount importance.

Conventional lipid management for high‐risk cardiovascular patients involves a comprehensive treatment process encompassing pharmacotherapy, lifestyle intervention, and patient self‐care, which can reduce cardiovascular event risk but often lacks specificity and individualization [6]. The humanistic nursing concept, rooted in person‐centered philosophy, places the patient at the core of care delivery by emphasizing respect for individual differences, emotional support, and the promotion of patient autonomy and dignity. In practice, this approach translates to individualized care plans that address not only the biomedical aspects of disease but also the psychological, social, and spiritual dimensions of the patient experience [7, 8]. Cardiovascular specialty care, meanwhile, provides structured, evidence‐based nursing services tailored to patients with cardiovascular conditions, including close monitoring of clinical parameters, medication management, dietary guidance, physical activity prescription, and comprehensive health education [9]. Integrating the humanistic concept into cardiovascular specialty care aims to deliver professional, patient‐centered services while attending to patients' emotional needs, thereby fostering a supportive therapeutic environment that may enhance clinical outcomes [10, 11]. However, empirical evidence regarding the clinical effectiveness of this integrated approach in high‐risk cardiovascular patients remains limited.

Based on this background, the present study was designed to retrospectively analyze the potential associations of cardiovascular specialty care based on the humanistic concept with medication adherence, glycolipid metabolism, psychological status, self‐management ability, and quality of life in high‐risk cardiovascular patients, with the aim of providing evidence for the application of humanistic nursing in cardiovascular disease management.

## 2 Materials and Methods

### 2.1 Statement of Ethics

This study was approved by the Ethics Committee of China‐Japan Friendship Hospital (Approval No. 2023‐KY‐315). Given the retrospective design of this study, which exclusively utilized de‐identified data extracted from existing medical records, the Ethics Committee granted a waiver of individual informed consent in accordance with the Declaration of Helsinki and national regulatory guidelines governing retrospective research. This waiver was justified on the grounds that the study posed no more than minimal risk to participants, that obtaining consent was impracticable given the retrospective nature of data collection, and that all data were handled with strict confidentiality protections. Patient privacy was safeguarded through anonymization of all identifiable information prior to analysis.

### 2.2 Study Design

This retrospective analysis examined 115 patients with high‐risk cardiovascular disease who were treated at our institution between December 2023 and November 2024. Patient data, including demographic characteristics, laboratory results, and standardized assessment scores, were extracted from the electronic medical record system. During their hospitalization and follow‐up period, all patients had undergone standardized assessments as part of routine clinical practice: medication adherence was evaluated using the MMAS‐8, psychological status was assessed using the STAI, self‐management ability was measured using the ESCA scale, and quality of life was assessed using the GQOLI‐74 questionnaire. These assessments were administered by trained nursing staff at admission and at the end of the intervention period (typically at discharge or the final follow‐up visit) as part of the hospital's standard clinical care protocol. Patients were divided into two groups based on the care regimen documented in their records: the control group (*n* = 55, routine lipid care) and the observation group (*n* = 60, cardiovascular specialty care based on the humanistic concept). Patient selection was based on the completeness of medical records; all patients who met the inclusion criteria and had complete pre‐ and post‐intervention assessment data during the study period were included.

### 2.3 Inclusion and Exclusion Criteria

Inclusion criteria were as follows: (1) meeting the diagnostic criteria for high‐risk cardiovascular disease as formulated by the World Health Organization [12], defined as the presence of three or more of the following risk factors: hypertension, diabetes mellitus, dyslipidemia, current smoking, obesity (body mass index ≥ 28 kg/m²), or a family history of premature cardiovascular disease, or having established atherosclerotic cardiovascular disease with a predicted 10‐year cardiovascular event risk exceeding 20% as confirmed by electrocardiogram, cardiac ultrasound, and relevant clinical examinations; (2) age > 18 years; (3) complete clinical data including pre‐ and post‐intervention standardized assessment scores; (4) absence of serious neurological diseases or cognitive dysfunction; (5) normal communication ability.

Exclusion criteria were as follows: (1) presence of serious cardiac arrhythmia or aortic coarctation; (2) hepatic, renal, or other organ insufficiency; (3) prior history of cardiovascular or cerebrovascular events requiring acute intervention; (4) hematological system diseases; (5) congenital immune system disorders.

### 2.4 Control Group

The control group received routine lipid management, which included the following components: close monitoring of blood lipid levels with administration of lipid‐lowering medications under physician guidance, attention to drug side effects, and regular review of relevant laboratory indicators; dietary counseling following the principles of low‐fat, low‐cholesterol, low‐sugar, and high‐fiber intake, with emphasis on increased consumption of vegetables and fruits, reduction of fried foods, and limitation of saturated and trans fatty acid intake; guidance on aerobic exercise such as walking and jogging, with exercise intensity individualized according to each patient's physical condition, targeting 30 min per session, 3–5 times per week; and routine health education covering disease‐related information, precautions, and the importance of regular hospital follow‐up. Dietary adherence was monitored through patient self‐reported dietary logs reviewed at each follow‐up visit, supplemented by nutritional counseling from clinical dietitians. To minimize confounding from occupational and lifestyle variations, patients in both groups received standardized instructions regarding daily activity levels, rest schedules, and work‐related physical exertion during the intervention period, and baseline occupational status was comparable between groups as confirmed by demographic analysis.

### 2.5 Observation Group

The observation group received cardiovascular specialty care based on the humanistic concept in addition to the routine care described above. The implementation process comprised six key components. First, a cardiovascular specialty nursing team was established, led by a head nurse and comprising five experienced cardiovascular nurses and one chief physician serving as therapeutic director. Team members underwent systematic training in cardiovascular pathophysiology, pharmacology, specialized nursing operations, clinical assessment, emergency management, communication skills, psychological support, and professional attributes including empathy and adaptability, followed by competency assessment and certification. Second, upon admission, nursing staff greeted patients warmly, provided self‐introductions to establish rapport, assessed patient needs and clinical severity, and oriented patients to the ward environment, facilities, and departmental regulations. Third, general nursing care included regular ward cleaning and disinfection, individualized dietary arrangements considering patient conditions, ethnic preferences, and dietary habits with coordination with the nutrition department, disease education covering causes, risks, precautions, and medication effects, and encouragement of medication adherence through sharing of successful patient experiences. Fourth, therapeutic nursing involved strict adherence to physician orders for medication administration with attention to drug action characteristics, infusion rate adjustment, monitoring of drug effects and adverse reactions, and clear explanation of all nursing procedures to patients. Fifth, psychological nursing was delivered through daily communication sessions (approximately 30 min) between the responsible nurse and each patient to assess psychological status, disease knowledge, and treatment compliance, with targeted interventions including affirmation of patients' social and family contributions to enhance self‐worth, and adaptation of disease education language to patients' educational levels. Sixth, discharge care included completion of nursing satisfaction questionnaires, collection of patient and family contact information, establishment of a WeChat communication group, regular telephone follow‐up, and weekly dissemination of educational materials on cardiovascular disease management, diet, exercise, and follow‐up schedules through text, images, and videos.

### 2.6 Medication Adherence

Medication adherence was assessed using the Morisky Medication Adherence Scale (MMAS‐8) [13], a validated 8‐item self‐report questionnaire. Each item was scored from 0 to 1, yielding a total score ranging from 0 to 8. Scores below 6 indicated poor adherence, scores of 6–7 indicated moderate adherence, and a score of 8 indicated good adherence. Higher total scores reflected better medication adherence.

### 2.7 Glycolipid Metabolism Parameters

Fasting venous blood samples were collected from all patients in the morning. Serum was separated by centrifugation (model: LL900, Luoyang Hongshi Machinery Equipment Co. Ltd.) at 3000 r/min for 10 min (centrifugal radius 10 cm). Blood glucose indicators, including glycated hemoglobin (HbA1c), fasting plasma glucose (FPG), and 2‐h postprandial glucose (2hPG), as well as lipid indicators including triglycerides (TG), total cholesterol (TC), and low‐density lipoprotein cholesterol (LDL‐C), were measured using an automated biochemistry analyzer (Hitachi 7600, Direx Medical Technology Co. Ltd.).

### 2.8 Psychological Status

Psychological status was assessed using the State‐Trait Anxiety Inventory (STAI) [14], a widely used and well‐validated self‐report instrument for measuring anxiety in clinical populations. The STAI comprises 40 items divided into two subscales of 20 items each. The State Anxiety Inventory (S‐AI) measures transient anxiety, reflecting the intensity of current feelings of tension, nervousness, worry, and apprehension at a particular moment. The Trait Anxiety Inventory (T‐AI) measures stable, dispositional anxiety, reflecting the individual's general tendency to perceive situations as threatening and to respond with elevated anxiety. Each item is rated on a 4‐point Likert scale (1 = almost never to 4 = almost always), with total scores for each subscale ranging from 20 to 80. Higher S‐AI scores indicate greater current anxiety severity, while higher T‐AI scores indicate a stronger habitual tendency toward anxiety. The STAI has demonstrated strong psychometric properties in cardiovascular populations, with Cronbach's alpha coefficients of 0.90 and 0.91 for the S‐AI and T‐AI subscales, respectively [14].

### 2.9 Self‐Management Ability

Self‐management ability was assessed using the Exercise of Self‐Care Agency Scale (ESCA) [15], which evaluates patients' capacity for self‐directed health management across four dimensions: self‐concept (8 items, 40 points), which reflects patients' perception of their own health status and self‐worth; self‐care responsibility (8 items, 40 points), which assesses patients' sense of accountability for their own health behaviors; self‐care skills (13 items, 52 points), which measures patients' ability to perform specific health‐related activities; and health knowledge (8 items, 40 points), which evaluates patients' understanding of disease and health management principles. The total score is 172 points, with higher scores indicating greater self‐management ability.

### 2.10 Quality of Life

Quality of life was assessed using the Generic Quality of Life Inventory‐74 (GQOLI‐74) [16], a comprehensive, multidimensional instrument developed and validated for the Chinese population that evaluates overall well‐being across four domains. The material life domain assesses satisfaction with economic status, housing conditions, community services, and living environment. The somatic function domain evaluates physical health status, including the ability to perform daily activities, sleep quality, appetite, energy levels, and the presence or absence of physical pain and discomfort. The social function domain measures social integration, interpersonal relationships, social support, work capacity, and recreational activities. The psychological function domain assesses emotional well‐being, including self‐esteem, cognitive function, mood stability, and overall life satisfaction. Each domain is scored from 0 to 100 points, with higher scores indicating better quality of life. The GQOLI‐74 has demonstrated good reliability and validity in Chinese cardiovascular populations, making it well‐suited for assessing quality of life in the present study population.

### 2.11 Sample Size Estimation

Given the retrospective design of this study, a formal a priori sample size calculation was not performed. However, a post‐hoc power analysis was conducted using G*Power 3.1 software to evaluate the statistical adequacy of the sample. Based on the observed difference in total medication adherence between the observation group (95.00%) and the control group (81.82%), with a total sample of 115 patients (55 in the control group and 60 in the observation group), the study achieved a statistical power of 0.81 at a two‐sided significance level of 0.05 (Cohen's w = 0.26). For the primary continuous outcomes, based on the observed effect sizes for post‐intervention glycolipid metabolism parameters (mean Cohen's d ranging from 0.49 to 0.58), the achieved power ranged from 0.78 to 0.88. These results suggest that the sample size was adequate to detect clinically meaningful differences for the primary outcomes, although the power may have been limited for detecting smaller effect sizes. This limitation is acknowledged, and future prospective studies with larger sample sizes and a priori power calculations are warranted to confirm these findings.

### 2.12 Statistical Processing

SPSS 25.0 statistical software was used for data analysis. The normality of continuous variables was assessed using the Shapiro‐Wilk test, supplemented by visual inspection of Q‐Q plots and histograms. All continuous variables in this study, including glycolipid metabolism parameters (HbA1c, FPG, 2hPG, TG, TC, LDL‐C), anxiety scores (S‐AI, T‐AI), self‐management scores (ESCA subscales), and quality of life scores (GQOLI‐74 domains), were found to follow an approximately normal distribution (all Shapiro‐Wilk *p* > 0.05). Accordingly, measurement data conforming to normal distribution were expressed as mean ± standard deviation ($\bar{x}\pm$ s) and compared using the independent‐samples *t*‐test. Count data were expressed as *n* (%) and compared using the chi‐square (χ²) test. Differences were considered statistically significant at *p* < 0.05. All reported P‐values were two‐sided. We acknowledge that given the multiple comparisons performed in this study, the probability of type I error may be inflated, and readers should interpret individual *p*‐values with appropriate caution.

## 3 Results

### 3.1 Comparison of Baseline Clinical Data Between the Two Groups

The two groups were comparable in terms of age, sex, body mass index, prevalence of hypertension, coronary heart disease, educational level, dietary habits (high‐fat, high‐sugar, high‐salt diet), and physical activity (aerobic exercise 1–2 times per week), with no statistically significant differences (all *p* > 0.05), as shown in Table 1.

**Table 1:** Comparison of baseline clinical data between the two groups.

Variable		Control group (*n* = 55)	Observation group (*n* = 60)	χ²/t	*p*
Age (years)		69.63 ± 7.12	69.88 ± 7.10	0.188	0.851
Sex (*n*, %)	Male	35 (63.64)	39 (65.00)	0.023	0.879
	Female	20 (36.36)	21 (35.00)		
BMI (kg/m²)		25.75 ± 2.82	25.89 ± 2.95	0.260	0.796
Hypertension (*n*, %)	Yes	31 (56.36)	35 (58.33)	0.046	0.831
	No	24 (43.64)	25 (41.67)		
Coronary heart disease (*n*, %)	Yes	24 (43.64)	25 (41.67)	0.046	0.831
	No	31 (56.36)	35 (58.33)		
Educational level (*n*, %)	≤ Junior high school	32 (58.18)	38 (63.33)	0.320	0.572
	≥ High school	23 (41.82)	22 (36.67)		
High‐fat/sugar/salt diet (*n*, %)	Yes	40 (72.73)	46 (76.67)	0.236	0.627
	No	15 (27.27)	14 (23.33)		
Aerobic exercise 1–2 times/week (*n*, %)	Yes	37 (67.27)	40 (66.67)	0.005	0.945
	No	18 (32.73)	20 (33.33)		

### 3.2 Comparison of Medication Adherence between the Two Groups

The total medication adherence rate in the observation group (95.00%) was significantly higher than that in the control group (81.82%), and this difference was statistically significant (χ²=4.973, *p* = 0.026), suggesting that cardiovascular specialty nursing based on the humanistic concept was associated with improved medication adherence, as shown in Table 2.

**Table 2:** Comparison of medication adherence between the two groups (*n*, %).

Category	Control group (*n* = 55)	Observation group (*n* = 60)	*χ*²	*p*
Good adherence	30 (54.55)	35 (58.33)		
Moderate adherence	15 (27.27)	22 (36.67)		
Poor adherence	10 (18.18)	3 (5.00)		
Total adherence rate	45 (81.82)	57 (95.00)	4.973	0.026

### 3.3 Comparison of Glycolipid Metabolism Parameters Between the Two Groups

Glycolipid metabolism parameters serve as important clinical indicators for evaluating glucose and lipid homeostasis. HbA1c, FPG, and 2hPG reflect glycemic control, while TG, TC, and LDL‐C are established markers of lipid metabolism. Before the intervention, there were no statistically significant differences between the two groups in any glycolipid metabolism parameter (all *p* > 0.05), as shown in Table 3.

**Table 3:** Comparison of glycolipid metabolism parameters between the two groups before nursing ($\bar{x}\pm$ s).

Parameter	Control group (*n* = 55)	Observation group (*n* = 60)	*t*	*p*
HbA1c (%)	7.19 ± 1.26	7.27 ± 1.29	0.336	0.738
FPG (mmol/L)	6.75 ± 1.25	6.78 ± 1.31	0.125	0.900
2hPG (mmol/L)	10.49 ± 1.27	10.52 ± 1.32	0.124	0.902
TG (mmol/L)	2.55 ± 0.28	2.59 ± 0.30	0.737	0.462
TC (mmol/L)	8.11 ± 1.57	8.06 ± 1.49	0.175	0.861
LDL‐C (mmol/L)	5.52 ± 1.63	5.55 ± 1.65	0.098	0.922

2hPG = 2‐h postprandial glucose, FPG = fasting plasma glucose, HbA1c = glycated hemoglobin, LDL‐C = low‐density lipoprotein cholesterol, TC = total cholesterol, TG = triglycerides.

**Figure 1:**
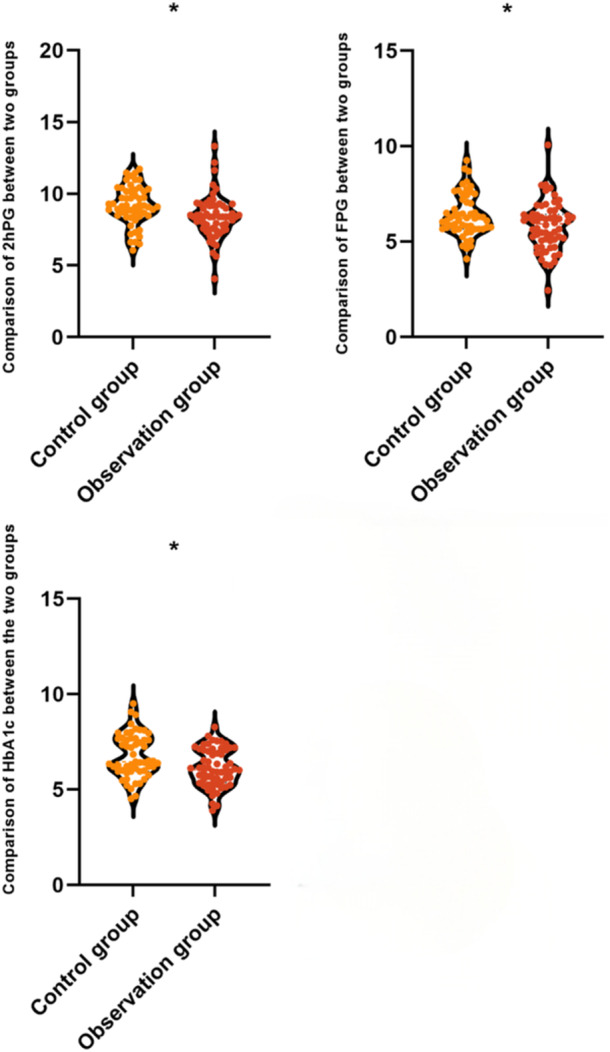
Comparison of glucose metabolism levels between the two groups after nursing.

**Figure 2:**
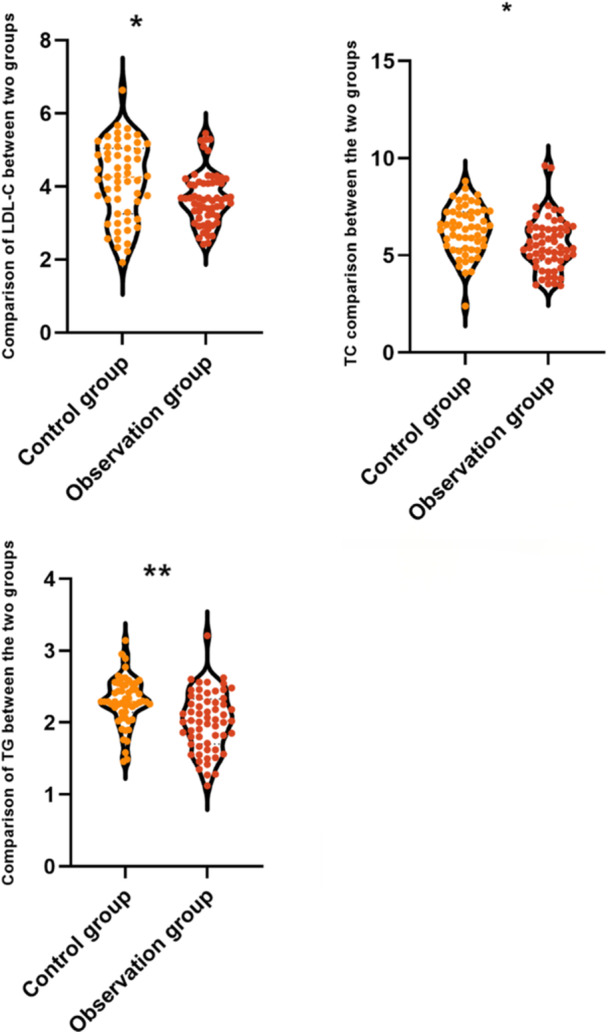
Comparison of lipid metabolism levels between the two groups after nursing. 2hPG = 2‐h postprandial glucose; FPG = fasting plasma glucose; HbA1c = glycated hemoglobin; LDL‐C = low‐density lipoprotein cholesterol; TC = total cholesterol; TG = triglycerides; * *p* < 0.05, **p < 0.001.

After the intervention, all glycolipid metabolism indicators were significantly lower in the observation group compared with the control group: HbA1c (6.14 ± 0.98 vs. 6.79 ± 1.18, *t* = 3.138, *p* = 0.002), FPG (5.81 ± 1.28 vs. 6.38 ± 1.11, *t* = 2.518, *p* = 0.013), 2hPG (8.39 ± 1.47 vs. 9.17 ± 1.34, *t* = 2.952, *p* = 0.004), TG (2.02 ± 0.41 vs. 2.29 ± 0.33, *t* = 3.823, *p* < 0.001), TC (5.59 ± 1.34 vs. 6.30 ± 1.25, *t* = 2.881, *p* = 0.005), and LDL‐C (3.68 ± 0.72 vs. 4.14 ± 1.06, *t* = 2.672, *p* = 0.009). These results suggest that cardiovascular specialty care based on the humanistic concept was associated with improved glycolipid metabolism, as shown in Figures 1 and 2.

### 3.4 Comparison of Psychological Status Between the Two Groups

Before the intervention, there were no statistically significant differences between the two groups in S‐AI scores (40.88 ± 4.63 vs. 40.85 ± 4.60, *t* = 0.035, *p* = 0.972) or T‐AI scores (40.78 ± 4.71 vs. 40.80 ± 4.72, *t* = 0.023, *p* = 0.982), as shown in Table 4.

**Table 4:** Comparison of psychological status between the two groups before nursing (scores, $\bar{x}\pm$ s).

Parameter	Control group (*n* = 55)	Observation group (*n* = 60)	*t*	*p*
S‐AI	40.88 ± 4.63	40.85 ± 4.60	0.035	0.972
T‐AI	40.78 ± 4.71	40.80 ± 4.72	0.023	0.982

Abbreviations: S‐AI, State Anxiety Inventory; T‐AI, Trait Anxiety Inventory.

**Figure 3:**
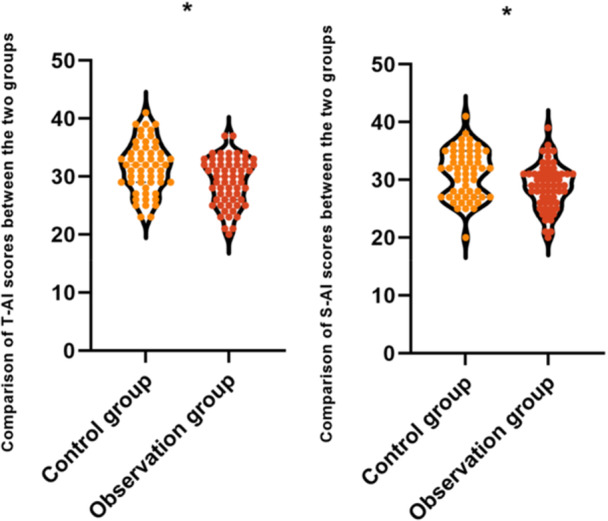
Comparison of psychological status between the two groups after nursing. S‐AI = State Anxiety Inventory; T‐AI = Trait Anxiety Inventory; *p < 0.05.

After the intervention, the observation group demonstrated significantly lower S‐AI scores (28.88 ± 3.90 vs. 31.12 ± 4.21, *t* = 2.924, *p* = 0.004) and T‐AI scores (29.17 ± 4.02 vs. 31.65 ± 4.35, *t* = 3.127, *p* = 0.002) compared with the control group, suggesting that the humanistic concept‐based cardiovascular specialty care was associated with reduced anxiety levels, as shown in Figure 3.

### 3.5 Comparison of Self‐Management Ability Between the Two Groups

Before the intervention, there were no statistically significant differences between the two groups in any ESCA subscale scores: self‐concept (12.58 ± 1.32 vs. 12.66 ± 1.27, *t* = 0.290, *p* = 0.773), self‐care responsibility (15.31 ± 1.56 vs. 15.46 ± 1.57, t = 0.513, *p* = 0.609), self‐care skills (21.44 ± 2.18 vs. 21.31 ± 2.25, *t* = 0.314, *p* = 0.754), and health knowledge (17.13 ± 2.17 vs. 17.08 ± 2.12, *t* = 0.100, *p* = 0.921), as shown in Table 5.

**Table 5:** Comparison of self‐management ability between the two groups before nursing (scores, $\bar{x}\pm$ s).

ESCA Domain	Control group (*n* = 55)	Observation group (*n* = 60)	*t*	*p*
Self‐concept	12.58 ± 1.32	12.66 ± 1.27	0.290	0.773
Self‐care responsibility	15.31 ± 1.56	15.46 ± 1.57	0.513	0.609
Self‐care skills	21.44 ± 2.18	21.31 ± 2.25	0.314	0.754
Health knowledge	17.13 ± 2.17	17.08 ± 2.12	0.100	0.921

**Figure 4:**
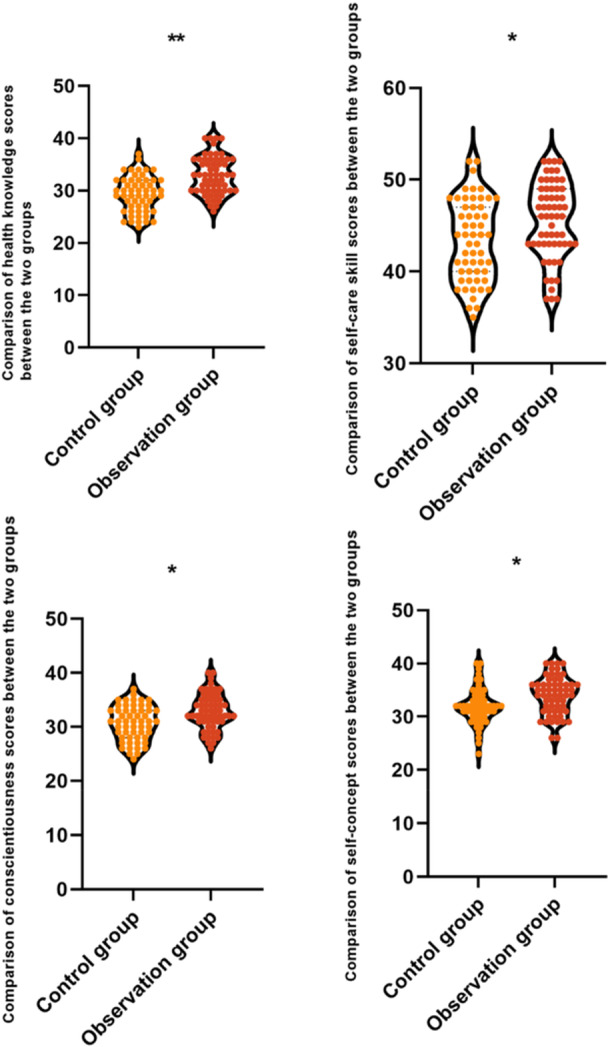
Comparison of self‐management ability between the two groups after nursing. *Note:* *p < 0.05, **p < 0.001.

After the intervention, the observation group showed significantly higher scores than the control group in all ESCA domains: self‐concept (33.98 ± 3.51 vs. 31.97 ± 3.44, *t* = 3.069, *p* = 0.003), self‐care responsibility (32.83 ± 3.31 vs. 30.68 ± 3.21, *t* = 3.460, *p* < 0.001), self‐care skills (45.54 ± 4.72 vs. 43.36 ± 4.41, *t* = 2.519, *p* = 0.013), and health knowledge (32.92 ± 3.61 vs. 29.29 ± 3.42, *t* = 5.452, *p* < 0.001), suggesting that the intervention was associated with improved self‐management ability, as shown in Figure 4.

### 3.6 Comparison of Quality of Life Between the Two Groups

Before the intervention, there were no statistically significant differences between the two groups in any GQOLI‐74 domain: material life (62.23 ± 6.61 vs. 62.28 ± 6.58, *t* = 0.041, *p* = 0.968), somatic function (58.21 ± 6.48 vs. 58.59 ± 6.52, *t* = 0.313, *p* = 0.755), social function (61.37 ± 6.21 vs. 61.79 ± 6.20, *t* = 0.363, *p* = 0.718), and psychological function (60.31 ± 6.48 vs. 60.35 ± 6.51, *t* = 0.033, *p* = 0.974), as shown in Table 6.

**Table 6:** Comparison of quality of life between the two groups before nursing (scores, $\bar{x}\pm$ s).

GQOLI‐74 Domain	Control group (*n* = 55)	Observation group (*n* = 60)	*t*	*p*
Material life	62.23 ± 6.61	62.28 ± 6.58	0.041	0.968
Somatic function	58.21 ± 6.48	58.59 ± 6.52	0.313	0.755
Social function	61.37 ± 6.21	61.79 ± 6.20	0.363	0.718
Psychological function	60.31 ± 6.48	60.35 ± 6.51	0.033	0.974

**Figure 5:**
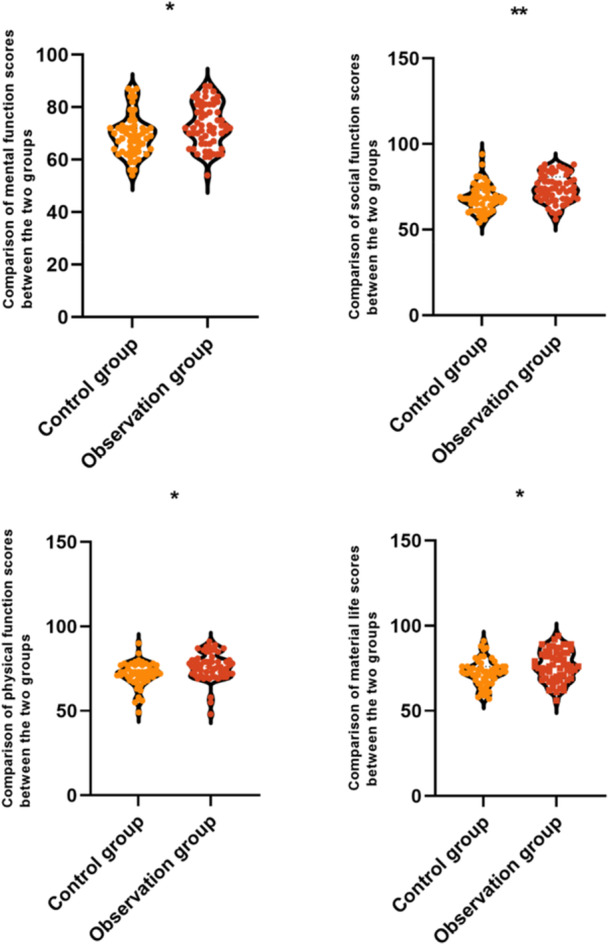
Comparison of quality of life between the two groups after nursing. *p < 0.05, **p < 0.001.

After the intervention, the observation group showed significantly higher scores than the control group in all GQOLI‐74 domains: material life (76.42 ± 8.92 vs. 72.21 ± 7.65, *t* = 2.700, *p* = 0.008), somatic function (76.15 ± 8.02 vs. 71.53 ± 7.28, *t* = 3.200, *p* = 0.002), social function (74.12 ± 8.11 vs. 68.52 ± 7.89, *t* = 3.693, *p* < 0.001), and psychological function (73.25 ± 8.21 vs. 69.21 ± 7.91, *t* = 2.641, *p* = 0.010), suggesting that the intervention was associated with improved quality of life, as shown in Figure 5.

## 4 Discussion

The present study demonstrated that cardiovascular specialty care based on the humanistic concept was associated with significantly higher medication adherence (95.00% vs. 81.82%), improved glycolipid metabolism parameters, reduced anxiety levels (both S‐AI and T‐AI), enhanced self‐management ability across all ESCA domains, and better quality of life across all GQOLI‐74 dimensions in high‐risk cardiovascular patients compared with routine care. These findings suggest that integrating humanistic principles into cardiovascular specialty nursing may represent a beneficial approach for the comprehensive management of this patient population.

Regarding medication adherence, our finding that the humanistic concept‐based nursing group showed a total adherence rate of 95.00% compared with 81.82% in the conventional care group is broadly consistent with published evidence indicating that structured cardiovascular rehabilitation, patient education, and sustained follow‐up support can improve adherence‐related behaviors. Aamot et al. [17] reported satisfactory long‐term adherence after a high‐intensity interval training cardiac rehabilitation program in patients with coronary artery disease. In addition, Burnier [18] emphasized that poor persistence and inadequate day‐to‐day execution of prescribed regimens are major barriers to blood pressure control in patients with hypertension, underscoring the importance of adherence‐focused management strategies. In the present study, the improvement in adherence may be attributed to the multi‐component nature of the intervention, which included disease education tailored to individual literacy levels, sharing of successful patient experiences to build confidence, and ongoing post‐discharge support through telephone follow‐up and WeChat‐based communication. These elements likely enhanced patients' understanding of the importance of medication and their motivation to adhere to prescribed regimens.

The observed improvements in self‐management ability across all four ESCA domains are also consistent with prior literature. Liu et al. [19] reported that humanistic care combined with structured health education significantly improved ESCA scores in patients with chronic heart failure, with the most pronounced improvements observed in the health knowledge and self‐care skills domains. Wang et al. [20] similarly demonstrated that patient‐centered nursing interventions emphasizing empowerment and self‐efficacy enhancement were associated with higher self‐management scores in cardiovascular patients compared with standard care. In our study, the comprehensive training of the nursing team in communication skills, empathy, and psychological support likely contributed to more effective patient education and empowerment, facilitating the development of self‐management behaviors.

With respect to psychological outcomes, our findings of significantly lower S‐AI and T‐AI scores in the observation group align with those of Wang et al. [21], who reported that humanistic care combined with predictive nursing reduced anxiety and depression scores in hemodialysis patients with cardiovascular comorbidities. Similarly, Chen et al. [22] reported that supportive non‐pharmacological intervention after cardiothoracic surgery was associated with reduced postoperative pain and anxiety, supporting the value of psychosocial and environmental support measures in perioperative cardiovascular care. The mechanisms underlying anxiety reduction in our study likely involve multiple pathways: the creation of a calm, clean ward environment; establishment of trusting nurse‐patient relationships through sincere, respectful communication; provision of clear, accessible disease information to reduce uncertainty; and affirmation of patients' self‐worth and social contributions. These elements collectively address the cognitive, emotional, and social dimensions of anxiety that are commonly observed in high‐risk cardiovascular patients, who may experience anxiety related to disease severity, prognosis uncertainty, and medication side effects [23, 24].

The improvements in quality of life observed across all four GQOLI‐74 domains are noteworthy. Prior studies have reported similar benefits of humanistic nursing approaches. Zheng et al. [25] demonstrated that comprehensive nursing care incorporating patient‐centered principles improved overall quality of life in patients with chronic cardiovascular disease, with significant improvements in physical, psychological, and social domains. Our results extend these findings by demonstrating associations between humanistic concept‐based cardiovascular specialty care and quality of life improvements in a specifically defined high‐risk population. The observed improvements in material life scores may reflect increased patient satisfaction with healthcare services, while improvements in somatic function likely reflect the combined effects of better glycolipid control and reduced anxiety on physical well‐being. The improvements in social function and psychological function domains are consistent with the emphasis of the humanistic approach on addressing patients' social and emotional needs.

Regarding glycolipid metabolism, the observation group showed significantly lower HbA1c, FPG, 2hPG, TG, TC, and LDL‐C levels after the intervention compared with the control group. These findings are consistent with those of Hui et al. [26], who reported that daily humanistic care integrated into pharmaceutical management improved glycemic control in patients with type 2 diabetes. Wang et al. [27] also demonstrated that comprehensive nursing interventions emphasizing patient participation, lifestyle modification, and medication management were associated with improved lipid profiles in patients with cardiovascular risk factors. It should be noted that the observed improvements in glycolipid metabolism in our study likely reflect the combined effects of enhanced medication adherence, dietary compliance, exercise engagement, and reduced psychological distress rather than a direct pharmacological effect of the nursing intervention itself. The humanistic approach may have indirectly improved metabolic outcomes by enhancing patients' motivation and capacity for self‐management of modifiable risk factors. Future studies with more rigorous designs, such as randomized controlled trials with blinded outcome assessment, are needed to clarify the causal pathways linking humanistic nursing interventions to metabolic improvements.

This study has several limitations that should be acknowledged. First, the retrospective design limits the ability to establish causal relationships between the intervention and observed outcomes, and the findings should be interpreted as associations rather than causal effects. The absence of randomization may have introduced selection bias, as patients who received the humanistic concept‐based care may have differed from control patients in unmeasured characteristics. Second, the relatively small sample size of 115 patients from a single institution may limit the generalizability of the findings, although post‐hoc power analysis confirmed adequate statistical power for the primary outcomes. Third, the short follow‐up period precludes assessment of the long‐term sustainability of the observed improvements. Fourth, reliance on existing medical records may have introduced information bias due to incomplete or missing data. Fifth, a prospective, multicenter randomized controlled trial design would be preferable to establish the efficacy of this intervention model and is recommended for future research. Despite these limitations, the present findings provide preliminary evidence supporting the potential benefits of integrating humanistic principles into cardiovascular specialty nursing for high‐risk patients.

## 5 Conclusion

In summary, cardiovascular specialty care based on the humanistic concept was associated with improved medication adherence, enhanced self‐management ability, reduced anxiety levels, improved glycolipid metabolism parameters, and better quality of life in high‐risk cardiovascular patients. These findings suggest that integrating humanistic nursing principles into cardiovascular specialty care may represent a promising approach for comprehensive disease management. Future prospective, multicenter randomized controlled trials with larger sample sizes and longer follow‐up periods are warranted to confirm these findings and establish causal relationships.

## Author Contributions

Min Wang and Yonghui Xu were involved in the conception and design, and analysis and interpretation of the data. Huina Wang, Shurui Dou, and JJingjing Xu participated in the drafting of the paper and revising it critically for intellectual content. Fanfan Wang and Yan Xu were responsible for the final approval of the version to be published. All authors agree to be accountable for all aspects of the work.

## Ethics Statement

The study was approved by the Ethics Committee of China‐Japan Friendship Hospital (2023‐KY‐315).

## Conflicts of Interest

M.W. and Y.X. declare that they have no conflicts of interest.

## Data Availability

The datasets used and/or analyzed during the current study are available from the corresponding author on reasonable request.
